# S16. GLUTAMATERGIC NEUROMETABOLITE LEVELS IN PATIENTS WITH TREATMENT-RESISTANT SCHIZOPHRENIA: A CROSS-SECTIONAL 3T PROTON MRS STUDY

**DOI:** 10.1093/schbul/sby018.803

**Published:** 2018-04-01

**Authors:** Yusuke Iwata, Shinichiro Nakajima, Eric Plitman, Jun Ku Chung, Fernando Caravaggio, Julia Kim, Eric Brown, Nathan Chan, Parita Shah, Sofia Chavez, Philip Gerretsen, Masaru Mimura, Gary Remington, Ariel Graff-Guerrero

**Affiliations:** 1 Centre for Addiction and Mental Health; 2 Centre for Addiction and Mental Health, University of Toronto; 3 Keio University School of Medicine

## Abstract

**Background:**

In terms of response to antipsychotic treatment, patients with schizophrenia can be classified into three groups; (1) treatment-resistant patients who are clozapine (CLZ)-resistant (ultra treatment-resistant schizophrenia [UTRS]), (2) treatment-resistant patients who are CLZ-responsive (TRS), and (3) patients who respond to non-CLZ antipsychotics (treatment non-resistant schizophrenia [TnRS]). The aim of this study was to examine glutamatergic neurometabolite levels in these three patient groups, along with healthy controls (HCs), using proton magnetic resonance spectroscopy (1H-MRS).

**Methods:**

Glutamate (Glu) and glutamate+glutamine (Glx) levels were assessed in the associative striatum (Str), anterior cingulate cortex (ACC), and dorsolateral prefrontal cortex (DLPFC) using 3T 1H-MRS (PRESS, TE=35ms). Neurometabolite levels were corrected for cerebrospinal fluid proportion.

**Results:**

A total of 100 participants (26 UTRS, 27 TRS, 21 TnRS, and 26 HCs) were included in this study. Patients with UTRS showed higher Glx levels in the ACC compared to HCs (p=0.038). When patients with UTRS and TRS were combined into one group, this subset of patients showed higher Glu and Glx levels in the ACC compared to HCs (p=0.028 and p=0.023, respectively). There were no significant group differences in the Str or DLPFC.

**Discussion:**

Previous findings reporting higher glutamatergic levels in the ACC of patients with TRS may be mainly influenced by patients with CLZ non-responder. Higher ACC glutamatergic neurometabolite level may be a biological trait of resistance to the first-line antipsychotic treatment that is retained even after CLZ administration.

